# Triple burden of disease and out of pocket healthcare expenditure of women in India

**DOI:** 10.1371/journal.pone.0196835

**Published:** 2018-05-10

**Authors:** Laishram Ladusingh, Sanjay Kumar Mohanty, Melody Thangjam

**Affiliations:** 1 Department of Demography & Statistics, International Institute for Population Sciences, Mumbai, Maharashtra, India; 2 Department of Fertility Studies, International Institute for Population Sciences, Mumbai, Maharashtra, India; 3 International Institute for Population Sciences, Mumbai, Maharashtra, India; University of West London, UNITED KINGDOM

## Abstract

**Aim:**

Women, unlike men, are subjected to triple burden of disease, namely, non-communicable and communicable diseases and reproductive health related diseases. To assess prevalence of triple burden of disease of currently married women and to contrast out of pocket maternal care expenditure of these diseases in India.

**Subject and methods:**

This study uses nationally representative unit level data from the 71st round (2014) of the National Sample Survey Organisation. Descriptive statistics and bivariate analysis are employed to assess triple burden of diseases by background of currently married women. Mean out of pocket (OOP) expenditure for healthcare care by demographic and household characteristics of women are also compared by type of diseases. Two parts model is adopted for assessment of determents of out of pocket healthcare expenditure of women.

**Results:**

Overall medical and non-medical expenses of non–communicable disease are much higher than those of other disease and disability, reproductive health related and communicable diseases. OOP expenditure for treatment of non-communicable diseases, reproductive health and related diseases and other disease and disability are significantly higher than the inpatient treatment of communicable diseases and the differences are statistically significant.

**Conclusion:**

Out of pocket expenditure for treatment of non-communicable diseases is the highest, followed by that of other diseases & disability, then reproductive health related diseases and the least is for communicable diseases. OOP expenditures for maternal healthcare in private health facilities are much higher than in public health facilities regardless of types of disease. Women from households having insurance of any member spent less than that of women from households not having health insurance. There is an urgent need to expand the outreach of the public health system in India to rural areas.

## Introduction

One of the most important distinctions in burden of diseases between men and women is that women have an additional burden of reproductive health related diseases including pregnancy and childbirth. Thus women are burden with out of pocket (OOP) healthcare expenditure not only with non-communicable and communicable diseases but also with reproductive health related diseases, unlike their men counterpart. This has made women more disadvantageous than men in the road to health for all. Therefore it is important to realize such triple burden of diseases of women, comprehend their healthcare and assess the burden of out of pocket expenditure (OOPE) of women's healthcare.

Non-communicable diseases (NCD) including cancer, cardiovascular diseases, diabetes and chronic respiratory diseases engulfed the lives of 35 million a year worldwide of which 18 million are women [1]. Then Secretary General of United Nations, [2] described the global NCD epidemic as a “public health emergency in slow motion”. Out of the 10.2 million deaths estimated for India in 2004, 5.2 million were due to NCD and overall age-standardized mortality rate of NCD were 769 per 100 000 men and 602 per 100 000 women [3]. For men it was 56 percent and for women 100 percent respectively more than that in high-income countries in 2004 [4]. According to recent estimates of IHME [5] the deaths from cardiovascular diseases (CVD), chronic respiratory diseases (CRD), cancer and diabetes accounts for 21.1, 11.8, 6.7 and 2.2 percent of all deaths in 2010. Recognizing the growing burden of non-communicable diseases a number of programmes, namely, National Programme for Prevention and Control of Cancer, Diabetes, Cardiovascular Diseases and Stroke (NPCDCS), and the National Programme for Health Care of the Elderly (NPHCE) were launched on World Health Day 2013 [6]. Despite the persistent sizeable share of infectious and communicable diseases, counting for 30 percent of all disease burden only a few infectious diseases are prioritized in the vertical control programmes. Among the infectious diseases only the control of HIV and leprosy seems to be successful but not that of diseases such as tuberculosis, malaria, and visceral leishmaniasis [7]. India’s triple burden of disease for women other than NCD, infectious and communicable diseases includes reproductive health, child health and nutrition. India’s burden of reproductive health, and child health and nutrition is greater than that in any other country [8]. Haemorrhage, sepsis, abortion complications and hypertensive disorders are the leading causes of maternal deaths in India [9]. National Rural Health Mission (NRHM) the flagship programme of the Ministry of Health and Family Welfare [10] has been successful in reducing maternal mortality ratio (MMR) considerably but has failed to meet the goal of achieving of MMR of 100 per 100,000 live births. The foregoing oration highlights in particular the share of women’s health in the overall burden of disease in India but research focus is pre-occupied in signaling out maternal health. One of the objectives of this paper is to assess the triple burden of disease of currently married women in India to provide a clearer picture of women’s health. Despite the escalating burden of diseases India has been unable to allocate an adequate share of its gross domestic product (GDP) to health; it spends less than 5 per cent of its GDP on health, of which the government's share is only slightly more than 1 per cent [11]. Thus treatment and prevention of diseases largely remains unfunded and under the circumstances out of pocket (OOP) expenditure is the main means of meeting healthcare needs. Out of pocket expenditure has impoverished 10.1 and 6.2 percent of rural and urban households respectively in India [12] and impoverishment effect of OOP expenditure has also been evident from [13]. Empirical evidence supporting the increase in OOP expenditure for healthcare as a share of household consumption with the ability to pay across social and economic strata is found in [14]. Defining catastrophic health expenditure as more than 10 percent of total household income spent as OOP on health [15] have highlighted significantly high healthcare cost associated with depressive disorder among women.

During the period 1993–2012 it has been have found that the annual growth rate per capita household OOP health spending was 6.14 percent which is twice than that of household per capita expenditure [16]. OOP healthcare expenditure for decedents is much higher than those of survivors in case of institutional treatment confirming heavy financial burden for end of life care [17] High OOP health expenditure, inadequate human resources for health, limited access to quality healthcare services, imbalanced resource allocation and rising financial burden on households are the main hurdles to healthcare and equity in India [18]. The importance of community-based health insurance in protecting poor households from impoverishment is also suggested by [19] in particular for areas where institutional capacity weak. Assessing the role of CHI (Community Health Insurance) scheme in coping with catastrophic health expenditure (CHE), [20] have found that covering hospital cost by CHI reduced CHE by halved. From an assessment of impact of health insurance on OOP expenditure of women who underwent hysterectomy in Gujarat, it is found that compared to 7.2 percent among women having insurance only 4.0 percent of uninsured women had undergone hysterectomy [21]. It has also been revealed that Janani SurakshaYojana (JSY) a conditional cash transfer incentive for institutional delivery under the National Rural Health Mission has not only enhance healthcare utilization but also lessen the economic burden of OOP healthcare expenditure of women [22]. Evidence of high OOP maternal care expenditure in India is provided by [23, 24]. Most of the aforesaid studies on women’s health predominantly dealt with reproductive and sexual health and do not encompassed non-communicable, infectious and communicable diseases and other diseases and disability. It is, therefore, felt the need for a study encompassing the triple burden of disease of women, namely, non-communicable and communicable diseases and reproductive health-related diseases. Keeping this in view the paper attempts to fill the gap, first by assessing the triple burden of disease of women and second by contrasting the OOP maternal healthcare expenditure of currently married women in India for non-communicable and communicable diseases and reproductive health-related diseases. The outcome of the study shall serve as key policy inputs to relook at positioning of women’s healthcare public policy in the right perspective.

The paper is organized as follows: data and methods are described in the next section, this is followed by a section on results and discussion and final section provides conclusion drawn from the study.

## Data and methods

### Data

This study is based on unit level data of 66,036 currently married women from the latest 71^st^ round of the National Sample Survey Organization (NSSO) surveyed in 2014. It is a nationally representative cross-sectional survey and it has adopted multistage stratified sampling design for ensuring regional and social group representation. Detail on morbidities, healthcare utilization, out of pocket expenditure for physician consultation, medicines, clinical and pathological examinations, bed charges, public and private status of health facilities, outpatient and inpatient treatments of individuals were collected in the survey.

Besides household monthly per capita expenditure (MPCE) and socio-demographic particulars of household members were also included in the data. Details of NSSO 71^st^ round data can be obtain from http://164.100.34.62/index.php/catalog/161.

### Methods

The diseases considered in this study are as reported by respondents which may or by and large are diagnosed cases. Diseases are broadly categorized into non-communicable, communicable, other diseases and disability and reproductive health related diseases. List of diseases in each of the category are provided in supporting information file.

OOP inpatient healthcare expenditure incurred in the last one year by currently married women for treatment of non-communicable and, communicable diseases, other diseases and disability and reproductive health related diseases are considered as the independent variable for analysis. OOP healthcare expenditure is adjusted for insurance and compensations for healthcare received from any source. On the basis of literature review a number of covariates which have concomitant bearing on OOP healthcare expenditure are also taken into consideration in the analysis. Covariates based on literature review included are residence background [4, 12], monthly per capita expenditure (MPCE) quintiles [4, 11, 25], insurance status at the household level [19, 20, 21], public and private [4, 22, 25, 26] status of healthcare provider and age of women [5,12,15]. Residence is considered as it is a proxy for accessibility to healthcare facilities, capacity to pay for OOP healthcare expenditure is accounted by MPCE and no capture the fact that high OOP expenditure due to lack of awareness of having insurance it is included in the analysis. To a considerable the health status depends on age and it is important to control it in the analysis It is also a fact that OOP healthcare expenditure is unwarrantedly high as available public health facility is unable to cope with the growing demand for healthcare and people are forced to avail healthcare from private healthcare facilities.

Frequency distribution and descriptive statistics are used for assessment of burden of disease and disease wise out of pocket (OOP) expenditure of maternal healthcare expenditure. For multivariate analysis two-part model (TPM) is used keeping in view need for adjustment of affliction by zeros in OOP expenditure. It is briefly described as follow:

$$Prob\;\left(y_{i}>0\right)=\frac{\exp\left(\beta x\right)}{1+\exp\left(\beta x\right)}$$ (1)

where yi = 0 indicates that women has no OOP payment for healthcare. The second part of the model predicts level of the OOP expenditure for health care, conditional on non-zero value. Estimates of predicted expenditure can be estimated by multiplying probabilities from the first part of the TPM by expected levels from the second part as:

$$E\left(y_{i}|x_{i}\right)=Prob\left(y_{i}>0\right)E\left(y_{i}|x_{i};y_{i}>0\right)$$ (2)

In the second part of Eq (2), we have as dependent variable log-transformed OOP payment for health care, given the women incurred health expenditure. To provide a consistent estimates of the OOP payment in the original scale of measurement, that is, Indian Rupees (INR) in this study, the exponential predicted values are multiplied by Duan’s non-parametric smearing factor [27] Ø, where, Ø = 1/n$\sum_{i}^{n}exp\;(ei)$, e*i* = ln y_i_-x_i_$\hat{\beta }$

The expected OOP health care expenditure is then estimated as:

$$E\left(y_{i}|x_{i}\right)=Prob\left(y_{i}>0\right)E\left(y_{i}|x_{i};y_{i}>0\right)\phi$$ (3)

## Results

Burden of disease of currently married women by types of reported diseases in the last 365 days preceding the survey are shown in Table 1 by selected background characteristics of women. No significant rural-urban gap can be notice in the reported burden of non-communicable, and communicable diseases, reproductive health related diseases and other diseases & disability. The rural-urban differential in prevalence of communicable diseases is 2.4 versus 2.5, for non–communicable diseases is 2.4 versus 2.8, for reproductive health-related diseases is 24.5 versus 25.8 and for other diseases & disability is 3.0 versus 2.9 percent respectively in 2014. More than two-third of currently married women are free from these diseases in the last 365 days. What is noted is that one in four of women suffered from reproductive health and related diseases. Only about one-tenth of women regardless of place of residence have reported to have any of the other diseases. The prevalence of communicable and non-communicable diseases as reported for the last 365 days among older currently married women 35 years and above are 3.3 and 4.4 percent while for younger women in 15–24 and 25–34 years the corresponding prevalence rates of these diseases are 1.5 and 2.1 percents and 1.0 and 1.7 percents respectively. On the other hand younger women have a higher burden of reproductive health related diseases in the last 365 days. The prevalence rates of reproductive health related diseases among women in 15–24 and 25–34 years are 45.9 and 31.3 percent respectively as compared to 6.3 percent among older women 35 years and above. Reported prevalence rates of other diseases and disability also show increasing trend with age. The association between age and prevalence of diseases is significant at P<0.01. Household’s economic well-being is measured in terms of monthly per capita expenditure (MPCE) and categorized into five quintiles for the purpose of cross validation of association burden of diseases among women and household economic status. During the reference period of 365 days, prevalence rates of communicable, non-communicable and other diseases and disability among currently married women belonging to the most economically better-off (fifth MPCE quintile) households are 2.8, 3.3 and 3.6 percent respectively which are marginally higher than the corresponding prevalence rates of 1.8, 2.2 and 2.2 percent among the women from the poorest (first MPCE quintile) households. Reproductive health and related diseases among currently married women is higher among women from poorer households. Association of prevalence of diseases with the level of household economic well-being is found to be significant at P<0.01.Prevalence of communicable, non-communicable, other & disability and reproductive health related diseases among currently married women is invariant of status of any household member having households have insurance or not.

**Table 1:** Burden of communicable, non-communicable, other & disability and reproductive health related diseases by background characteristics among currently married women in India, 2014.

	Communicable disease	Non communicable disease	Reproductive health related disease	Other disease & disability	No disease	N
**Residence**						
Rural	2.5	2.4	24.5	3.0	67.5	37495
Urban	2.4	2.8	25.8	2.9	66.2	28541
**Age group**						
15–24	1.5	1.0	45.9	1.3	50.3	14353
25–34	2.1	1.7	31.3	2.4	62.6	26806
35+	3.3	4.4	6.3	4.7	81.3	24877
MPCE quintile*						
First	1.8	2.2	25.1	2.2	68.6	11458
Second	2.2	2.2	25.7	2.6	67.3	14091
Third	2.6	2.2	25.2	3.2	66.8	13071
Fourth	2.7	2.8	25.0	3.2	66.3	12960
Fifth	2.8	3.3	24.4	3.6	66.0	14456
**Insurance for any household member**						
No	2.4	2.4	23.8	2.9	68.5	5574
Yes	2.4	2.6	25.2	3.0	66.8	60462
**Total**	2.4	2.6	25.1	3.0	67.0	66036

Note—*MPCE: Monthly per capita expenditure

The importance of the aforesaid assessment of maternal burden of diseases has shown the contrast between women’s reproductive related diseases and other diseases. The consideration in this paper to include child birth and pregnancy related cases is to bring out additional healthcare needs of women affects OOP healthcare expenditure not only varies by diseases but also by residence, age, household economic status, type of health facility and household insurance status. Table 2 shows the mean annual OOP healthcare expenditure by diseases and background characteristics of currently married women in India. The economic burden for treatment of non-communicable diseases is the highest, followed by that of other diseases & disability, reproductive health related diseases communicable diseases. The average annual OOP expenditure for treatment of these diseases are Rs. 12,669 Rs. 10,783, Rs. 7,142 and Rs. 6,570 respectively. As far as the OOP expenditure for treatment of communicable and non-communicable diseases and other diseases and disability are concerned no significant rural-urban difference can be notice. The annual OOP expenditure for treatment of other diseases & disability in urban and rural are Rs. 10,601 and Rs. 10,878 respectively and is significantly different at P<0.01. The annual OOP expenditure for treatment of reproductive health related diseases in urban and rural are Rs. 10,149 and Rs. 6,027 respectively and this two is significantly different at P<0.01. OOP expenditure for treatment of communicable and non-communicable diseases do not vary much by age of the currently married women but that for inpatient treatment of other diseases & disability and reproductive health related diseases OOP expenditure significantly shoots up with age particularly for the later diseases, as the OOP annual expenditure for older women in 35 plus is Rs. 11,325 as compared to Rs. 10,232 for the former. OOP healthcare expenditure for treatment of currently married women regardless of the type of diseases also is found to depend on affording capacity of households as measured by household monthly per capita expenditure (MPCE).

**Table 2:** Annual out of pocket maternal healthcare expenditure (in INR) by type of diseases and background characteristics of currently married women in India, 2014.

	Annual expenditure (in INR)				
	Communicable diseases	Non-communicable diseases	Reproductive health related diseases	Other diseases & disability	All diseases
**Residence**					
Rural	6405	12585	6027	10878	6880
Urban	6940	12845	10149	10601	10192
**Age group**					
15–24	6878	12661	6347	8401	6518
25–34	6620	11690	7308	10625	7647
35+	6421	13022	10232	11325	10612
MPCE quintile*					
First	4682	11102	3850	8730	4566
Second	7312	11045	5271	9672	6024
Third	5445	13291	6641	9910	7262
Fourth	5892	12043	8662	10318	8879
Fifth	8931	14931	14018	13915	13691
**Type of health facility**					
Public	2615	7203	2629	4629	2963
Private	9785	16241	14276	13895	14055
**Insurance for any household member**					
No	7140	18800	6445	9312	7400
Yes	6528	12309	7200	10896	7850
**Total**	6570	12669	7142	10783	7817

Note: INR-Indian rupees,

*MPCE: Monthly per capita expenditure

For all diseases, annual OOP inpatient healthcare expenditure concomitantly increases by MPCE quintiles and the differences are found to be significant. An important noticeable feature of the association between OOP healthcare expenditure of currently married women and household MPCE is that the rate of escalation of OOP healthcare expenditure for treatment of reproductive health related diseases is much higher than even that for treatment of non-communicable diseases for women from economically better off households specially belonging to the fourth and fifth MPCE quintiles. The annual OOP expenditure for treatment of reproductive health related diseases of women from the fourth and the fifth MPCE quintile households are Rs. 8,662 and Rs. 14,018 as against the corresponding expenditures of Rs. 12,043 and Rs. 14,931 for treatment of non-communicable diseases. Seeking healthcare in private health facilities is exorbitantly expensive than utilization of public health facilities as OOP healthcare expenditure for treatment of communicable, non-communicable, other diseases & disability and reproductive health related diseases in private facilities are found to be 3.7, 2.3, 3.0 and 5.4 times respectively higher than utilization of public health facilities. There is no systematic pattern of association between OOP healthcare expenditure of currently married women and insurance status. In the case of treatment of non-communicable diseases annual OOP expenditure healthcare of women from households with any member having insurance is Rs. 18,800 as against Rs. 12,309 for women from households with no member having insurance. But for the treatment of reproductive health related diseases OOP expenditure is Rs 7,200 for women from households with any member having insurance as against Rs. 6,445 for women with from households with no member having insurance. Maternal healthcare cost disaggregated by doctor/surgeon fee, expenditure for medicine, diagnostic tests and non-medical expenses are shown in Table 3.Overall medical and non-medical expenses of non–communicable disease are much higher than those of other disease and disability, reproductive health related and communicable diseases. OOP expenditures for care in private health facilities are many times higher than in public health facilities regardless of types of disease. For treatment in public health facilities, mean medical expenses for women is Rs. 1749 for communicable disease as compared to Rs. 5175 for non-communicable, while the corresponding figures are Rs. 8444 and Rs 14097 respectively for women who were treated in private facilities. Mean medical expenses for public hospital is high for non-communicable disease followed by other disease and disability, reproductive health related disease and communicable disease. But it is the other way for private hospital inpatient mean medical expenditure is more for non-communicable disease with an average of Rs 14,097 followed by Rs 12,429 for reproductive health related disease, Rs 11,839 and Rs 8,444 for other disease and disability and communicable disease respectively. OOP expenditures towards surgeon fees, medical costs and other medical items for seeking treatment in private facilities are invariably higher than public facilities, but the gap between private and public facilities for each component of medical expenses is much wider for all types of disease. The mean surgeon fees for reproductive health related disease for treatment in private facilities is Rs. 4384 as compared to Rs. 120 for public facilities and for non-communicable, the corresponding main expenses are Rs 3591 and Rs. 297 respectively. The mean medicine costs for treatment of non-communicable diseases in private hospital facilities is Rs 4928 as compared to Rs. 2580 for those treated in public facilities. For reproductive health related disease, the mean medical costs for treatment in private and public facilities are Rs. 3467 and Rs. 870 respectively.

**Table 3:** Itemize mean annual OOP expenditure in last 365 days by type of diseases and health facilities in India.

Itemize expenditures	Communicable disease	Non communicable disease	Reproductive health related disease	Other disease & disability
	**Overall**			
Doctor/Surgeon fees	881	2289	1772	2455
Medicine	2225	4000	1877	3295
Diagnostic tests	1174	2464	1383	2037
Other medical expenditure	1524	2531	1377	1981
Total medical expenditure	5442	10571	5762	9042
Transport for patient	361	746	557	632
Other non-medical expense	889	1499	976	1314
Total non-medical expenditure	1128	2098	1380	1741
Total expenditure on health care	6570	12669	7142	10783
	**Public Health Facility**			
Doctor/Surgeon fees	68	297	120	525
Medicine	999	2580	870	1836
Diagnostic tests	727	1763	663	1068
Other medical expenditure	295	1307	282	531
Total medical expenditure	1749	5175	1544	3511
Transport for patient	309	714	473	471
Other non-medical expense	658	1502	751	779
Total non-medical expenditure	866	2028	1085	1118
Total expenditure on health care	2615	7203	2629	4629
	**Private Health Facility**			
Doctor/Surgeon fees	1543	3591	4384	3431
Medicine	3223	4928	3467	4032
Diagnostic tests	1410	2783	2025	2457
Other medical expenditure	2524	3331	3108	2714
Total medical expenditure	8444	14097	12429	11839
Transport for patient	404	766	684	715
Other non-medical expense	1071	1497	1324	1578
Total non-medical expenditure	1341	2144	1847	2056
Total expenditure on health care	9785	16241	14276	13895

**Fig 1:**
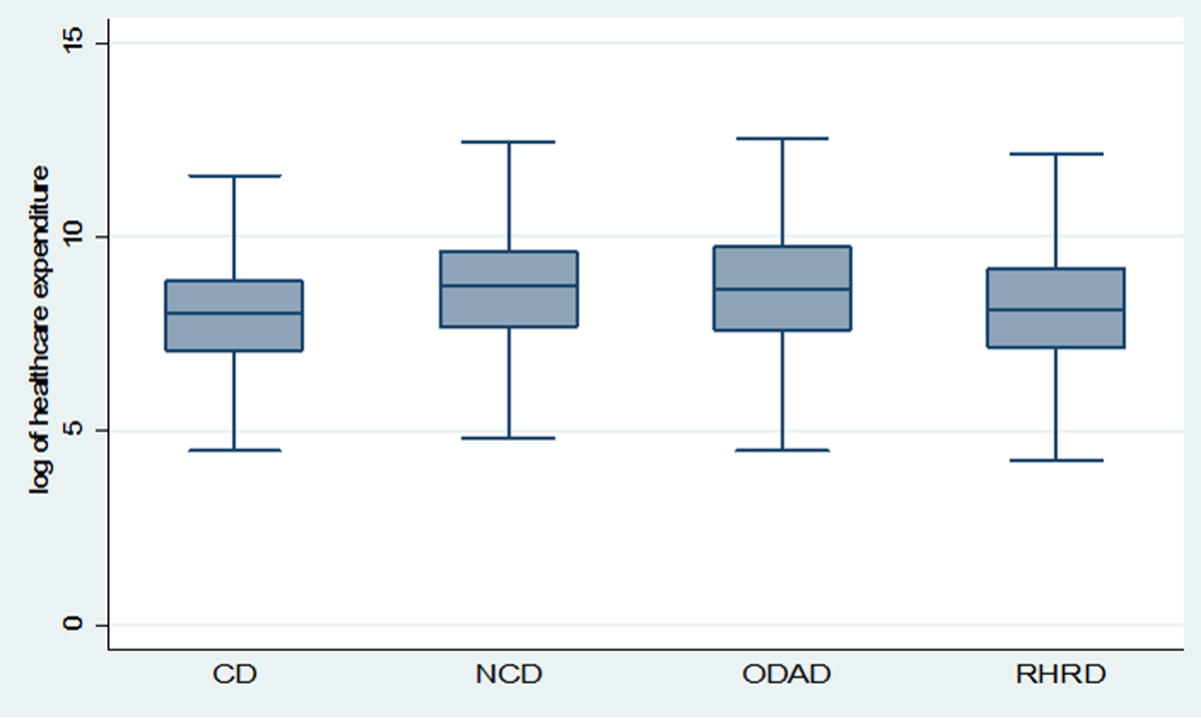
Box plot of annual OOP healthcare expenditure (log scale) by type of diseases. Note: CD–Communicable Disease, NCD- Non Communicable Disease, ODAD- Other disease & disability, RHRH- Reproductive health related disease.

Fig 1 shows the quartiles of mean annual OOP expenditure (in log scale) by type of diseases. It can be noted that in terms of median OOP healthcare expenditure it is highest for non-communicable diseases followed by reproductive health & related diseases, then other diseases & disability and communicable diseases. There is no significant variation in inter-quartile range of OOP healthcare expenditure by diseases each being close to 2 (in log scale).

**Fig 2:**
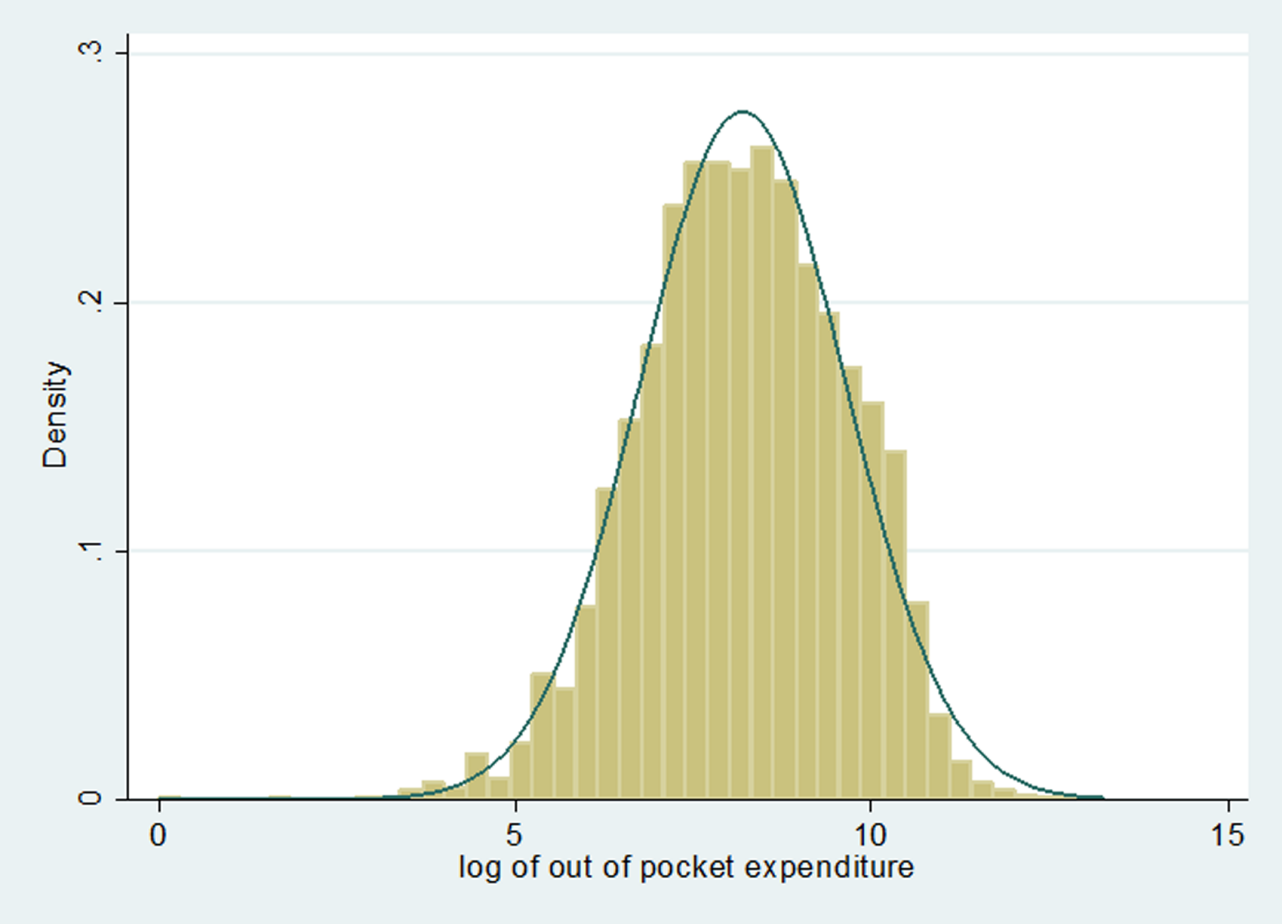
Histogram and density of out of pocket expenditure for healthcare (in log scale).

The distribution of out of pocket healthcare expenditure in log scale is shown in Fig 2 and estimate adjusted effects of background characteristics on out of pocket healthcare expenditure two part model is used. The distribution of log of out of pocket health expenditure follows normal distribution as noted from Fig 2 and justifies the appropriateness of considering multiple linear regression in the two part model.

Parameter estimates of out of pocket expenditure of women for two part model express in terms of odds ratios by taking antilogarithm of beta coefficients are shown in Table 4. The table includes odds ratios which indicate the magnitude and direction of difference in the OPP of each background characteristic in comparison to the reference category of characteristic, standard errors and 95% confidence interval of odds ratios.

**Table 4:** Estimates of coefficients of two part model for out of pocket expenditure of women in India, 2014.

			95% confidence Interval	
Background Characteristics	Odd ratio	Std. Err.	Lower	Higher
**Residence**				
Rural				
Urban	0.87^*^	0.02	0.84	0.90
**Age Group**				
15–24				
25–34	1.13^*^	0.02	1.09	1.17
35+	1.27^*^	0.03	1.21	1.34
MPCE quintile^*^				
First				
Second	1.21^*^	0.03	1.14	1.27
Third	1.32^*^	0.03	1.25	1.40
Fourth	1.48^*^	0.03	1.40	1.57
Fifth	1.66^*^	0.03	1.56	1.75
**Insurance status**				
No				
Yes	0.86^*^	0.03	0.81	0.91
**Type of health facility**				
Public				
Private	3.97^*^	0.02	3.83	4.11
**Disease category**				
CD				
NCD	1.63^*^	0.04	1.50	1.78
RHRD	1.53^*^	0.04	1.41	1.66
ODAD	1.47^*^	0.03	1.37	1.57

Note: CD–Communicable Disease, NCD- Non Communicable Disease, RHRH- Reproductive health related disease, ODAD- Other disease & disability

*significant at P < 0.01

It is noted that controlling for other factors, urban eta residents incurred 13 percent lower OOP healthcare expenditure than their rural counter parts and is statistically significant at P<0.01. This reflects better healthcare provisions and accessibility of healthcare in urban than in rural areas. Older women in 25–34 and 35 years and above incurred 13 and 27 percent higher out of pocket expenditure respectively than the younger in 15–24 years and the gap is statistically significant at P<0.01. Women from better off households as measure by higher monthly per capita expenditure (MPCE quintiles) adjusting for other covariates incurred higher OOP expenditure for maternal care confirming that those who have capacity to pay more for seeking treatment, it increases from 21 to 66 percent higher for women from households in second and fifth MPCE quintiles in comparison to that of women from households in the lowest MPCE quintile and are significant at P<0.01. Women from households with any member having insurance spent 16 percent less than women from households with no member having any insurance and the adjusted differential is statistically significant at P<0.01. Maternal healthcare in private health facility is 4 times more expensive than in public health facility. OOP expenditure for treatment of non-communicable diseases, reproductive health and related diseases and other disease and disability are respectively 63, 53 and 47 percent significantly higher than treatment of communicable diseases and the differences are significant at P<0.01. The differentials in mean OOP healthcare expenditure by backgrounds of women in Table 2 are in agreement with results of the multiple regression analysis.

## Conclusion

This study examines the triple burden of disease of currently married women and provide a holistic picture of women’s health and burden of out of pocket expenditure for maternal healthcare. The findings shall serve as key policy inputs. One-third of the currently married women reported any kind of disease, not even reproductive health related. The rural-urban gap in prevalence of communicable, non-communicable, reproductive health related diseases and other diseases & disability among currently married women in India for 2011–12 are 2.4 versus 2.5, 2.4 versus 2.8, 24.5 versus 25.8 and 3.0 versus 2.9 percent respectively, showing no significant rural-urban differential in burden of disease. This is suggestive of the need for equity in provisioning of healthcare facilities across the length and breadth of the country. Similar emphasis is also brought to focus by [26]. Reproductive health related diseases among currently married women is higher among women from poorer households. Association of prevalence of diseases by level of household economic well-being is found to be significant P<0.01. Thus healthcare system needs to reach out to underprivileged poor as poor women are more likely to ignore their health. The result is corroborates with findings of [25]. Prevalence of communicable, non-communicable, other & disability and reproductive health related diseases among currently married women is invariant of they belong to households with any member having insurance or not. The economic burden for treatment of non-communicable diseases is the highest, followed by that of other diseases & disability, then reproductive health related diseases communicable diseases and annual OOP expenditure for treatment of these diseases are Rs. 12,669 Rs. 10,783, Rs. 7,142 and Rs. 6,570 respectively. Non-communicable diseases are more chronic than other diseases and usually to be treated for longer time, making it more expensive than treatment of other diseases. As far as the OOP expenditure for treatment of communicable and non-communicable diseases and other diseases and disability are concerned no significant rural-urban difference can be notice. Overall medical and non-medical expenses of non–communicable disease are much higher than those of other disease and disability, reproductive health related and communicable diseases. Higher OOP expenditure for NCD than the other diseases among others is also found in [28]. OOP expenditures for seeking treatment from in private health facilities are many times higher than in public health facilities regardless of types of disease. Women from households with any member having insurance spent less than the women from households with no member having health insurance and the adjusted differential is statistically significant at P<0.01. The evidence of cushioning of OOP healthcare expenditure by insurance is also provided by [19, 20]. Maternal healthcare in private health facility is much more expensive than in public health facility. There is no doubt that facilities, services and appliances used in private health facilities are superior to those in public facilities. So the excessive medical expenses in private facilities are partly due to charges for these facilities. It is well known that economically better off patients and those who are willing to pay prefer to utilize health care from private facilities. OOP expenditure for treatment of non-communicable diseases, reproductive health and related diseases and other disease and disability are significantly higher than the inpatient treatment of communicable diseases and the difference is statistically significant.

Women’s healthcare program can take note of that burden of disease among married women emphasizing not only reproductive and sexual health but also non-communicable, infectious and communicable diseases and other diseases and disability. One of the limitations of this study is that the diseases considered in the study are as reported by the respondents and not diagnosed cases. The study also has not considered state and region contextual factors particularly health facilities adequacy and public health outreach programmes.

## Supporting information

S1 File Supporting information file. (DOCX)
