# Supplementary material for: Triple burden of disease and out of pocket healthcare expenditure of women in India
Source: PLoS One. 2018 May 10;13(5):e0196835. doi: 10.1371/journal.pone.0196835 (PMC5945049; doi:10.1371/journal.pone.0196835)
Supplement: S1 File — (DOCX) [file pone.0196835.s001.docx]

**S1. Supporting information**

| **Communicable Diseases** |
| --- |
| All other fevers (Includes malaria, typhoid and fevers of unknown origin, all specific fevers that do not have a confirmed diagnosis) |
| Diarrheas/ dysentery/ increased frequency of stools with or without blood and mucus in stools |
| Worms infestation |
| Fever due to Diphtheria, Whooping cough |
| Jaundice |
| Fever with loss of consciousness or altered consciousness |
| Tuberculosis |
| Fever with rash/ eruptive lesions |
| Filariasis |
| Tetanus |
| HIV/AIDS |
| Other sexually transmitted diseases |
| **Non-Communicable Diseases** |
| Hypertension |
| Heart disease: Chest pain, breathlessness |
| Diabetes |
| Bronchial asthma/ recurrent episode of wheezing and breathlessness with or without cough over long periods or known asthma) |
| Mental retardation |
| Mental disorders |
| Headache |
| Seizures or known epilepsy |
| Weakness in limb muscles and difficulty in movements |
| Stroke/ hemiplegia/ sudden onset weakness or loss of speech in half of body |
| Others including memory loss, confusion |
| Accidental injury, road traffic accidents and falls |
| Accidental drowning and submersion |
| Burns and corrosions |
| Poisoning |
| Cancers |
| Acute upper respiratory infections (cold, runny nose, sore throat with cough, allergic colds included) |
| Anaemia |
| Bleeding disorders |
| Under-nutrition |
| Goitre and other diseases of the thyroid |
| Others (including obesity) |
| Cough with sputum with or without fever and NOT diagnosed as TB |
| **Reproductive Health Related Diseases** |
| Any difficulty or abnormality in urination |
| Pain the pelvic region/reproductive tract infection/ Pain in male genital area |
| Change/irregularity in menstrual cycle or excessive bleeding/pain during menstruation and any other gynecological and anthological disorders female infertility |
| Pregnancy with complications before or during labour (abortion, ectopic pregnancy, abortion, hypertension, complications during labour) |
| Complications in mother after birth of child |
| Childbirth – Caesarean/ normal/ any other (for both live birth and stillbirth) |
| **Other Diseases and Disability** |
| Discomfort/pain in the eye with redness or swellings/ boils |
| Cataract |
| Glaucom |
| Decreased vision (chronic) NOT including where decreased vision is corrected with glasses |
| Others (including disorders of eye movements – strabismus, nystagmus, ptosis and adnexa |
| Earache with discharge/bleeding from ear/infections |
| Decreased hearing or loss of hearing |
| Diseases of mouth/teeth/gums |
| Pain in abdomen: Gastric and peptic ulcers/ acid reflux/ acute abdomen |
| Lump or fluid in abdomen or scrotum |
| Gastrointestinal bleeding |
| Skin infection (boil, abscess, itching) and other skin disease |
| Joint or bone disease/ pain or swelling in any of the joints, or swelling or pus from the bones |
| Back or body aches |
| Intentional self-harm |
| Assault |
| Contact with venomous/harm-causing animals and plants |
| Symptom not fitting into any of above categories |
| Could not even state the main symptom |
